# Trickle-Down Preferences: Preferential Conformity to High Status Peers in Fashion Choices

**DOI:** 10.1371/journal.pone.0153448

**Published:** 2016-05-04

**Authors:** Jeff Galak, Kurt Gray, Igor Elbert, Nina Strohminger

**Affiliations:** 1 Tepper School of Business, Carnegie Mellon University, Pittsburgh, Pennsylvania, United States of America; 2 Department of Psychology and Neuroscience, University of North Carolina, Chapel Hill, North Carolina, United States of America; 3 Independent Researcher, New York, New York, United States of America; 4 School of Management, Yale University, New Haven, Connecticut, United States of America; Taipei Veterans General Hospital, TAIWAN

## Abstract

How much do our choices represent stable inner preferences versus social conformity? We examine conformity and consistency in sartorial choices surrounding a common life event of new norm exposure: relocation. A large-scale dataset of individual purchases of women’s shoes (16,236 transactions) across five years and 2,007 women reveals a balance of conformity and consistency, moderated by changes in location socioeconomic status. Women conform to new local norms (i.e., average heel size) when moving to relatively higher status locations, but mostly ignore new local norms when moving to relatively lower status locations. In short, at periods of transition, it is the fashion norms of the rich that trickle down to consumers. These analyses provide the first naturalistic large-scale demonstration of the tension between psychological conformity and consistency, with real decisions in a highly visible context.

## Introduction

Conformity is undoubtedly a robust psychological phenomenon, but classic laboratory studies [1, 2] have difficulty speaking to the subtle variations we experience in everyday life. Qualitative and quantitative investigations into everyday conformity [3–6]—while informative—involve small sample sizes, specific locations, and external interventions, leaving open questions about large-scale endogenous conformity. Even recent large-scale network analyses of “spreading” tastes are often difficult to generalize beyond specific closed networks [7], leaving open questions about transitions between social networks.

To overcome the challenges of past research on everyday conformity, we examine changing preferences within a large dataset of real purchase behavior, obtained from an online retailer of luxury clothing brands. Clothing is a key expression of identity that balances conformity and self-expression [8, 9]. Sartorial choices, therefore, provide a convenient test case for how conformity plays out in real life. To study sartorial conformity, we examined a common life event where changes in clothing are especially visible: relocation. By looking at this life event, we can not only quantify overall conformity but also examine the impact of extrinsic social variables upon conformity. One important potential variable is socioeconomic status, such that conformity may depend upon the relative socioeconomic status between origin and destination.

Theories in sociology have argued that taste, especially for highly visible consumer products, flows from high status individuals to low status individuals [10]. This suggests that conformity should be more likely when an individual moves to an area higher in SES, in order to emulate high status others [11–13]. In contrast, when moving to areas with relatively lower status individuals, conformity may be less likely because these individuals may wish to maintain a sense of uniqueness by remaining consistent with their original preferences [14]. This tension between conformity and consistency is a hallmark of Optimal Distinctiveness Theory, which postulates that people balance fitting-in with remaining unique [15, 16]. However, we suggest a twist on Optimal Distinctiveness Theory, where people are motivated to fit in with high-status groups and remain unique from low-status groups. One way of investigating this potential asymmetry is through fashion choices.

Anecdotally, fashion does seem to progress from high status to low status: runway shows in Paris and Milan give way to celebrity wardrobes, then to clothing in high-end boutiques, before descending to malls, department stores and discount racks. These observations are echoed by the “upper class theory of fashion,” which predicts a trickle-down of preferences from the elite leisure class to the proletariat [17]. However, fashion and style can also be inspired from relatively low SES groups, such as the rise (and resurrection) of grunge, the popularity of tattoos, and the power of hip-hop fashions.

Although many have discussed questions of conformity versus consistency of sartorial preferences in light of SES disparities, no work has empirically examined the interplay of these issues. In the present work, we use large scale naturalistic data to investigate the interplay of conformity/consistency and relative SES for one particular sartorial preference, shoe heel-size. Although sartorial choices are multi-dimensional [18], shoe heel-size is a quantitative measure that varies across geographic locations [19]. Relocation provides an ideal lens to examine SES and conformity because it represents a relatively rapid and discrete change in SES environment. Specifically, we investigate whether post-relocation purchases stay consistent with past behavior, or whether they follow new destination norms—and whether conformity reflects the relative status of the new location.

## Materials and Method

### Dataset Summary

The dataset comes from an online retailer of luxury clothing brands. Data included 16,236 shoe purchases over five years (2010–2015) from 2,007 women who had changed primary residence locations (i.e., US Zip Code) at least once. Average heel-size of shoes purchased by participants in the destination location was the key dependent variable (i.e., *new sartorial behavior*). Key predictor variables were the average heel-size purchased by participants in the origin location (*past sartorial behavior*); the average heel-sizes purchased by others in the participant’s origin location (*origin norms*) and destination location (*destination norms*); and the median income level in the US Census Region of origin (*origin SES*) and destination (*destination SES*), as given by 2010 US Census data.

### Sample

Our data come from an online retailer of luxury clothing brands. The retailer’s name cannot be disclosed due to a non-disclosure agreement prohibiting the naming of the company, though use of the data for research purposes and disclosure of summary statistic is permitted. Of primary interest are individuals who we can identify as having moved from one geographic location to another during our observation window. This identification task was accomplished by defining a “mover” as someone in United States who had at least five transactions in one location followed by at least five transactions in a new location. Though this specification is imperfect, it is reasonable to assume that an individual who purchased clothing a number of times in one area and then systematically switched their purchases to a new area has, at minimum, a strong physical presence in the two geographies, and, at best, permanently moved. Moreover, because our measure of interest is heel size, we further restricted our data set to those individuals who purchased at least one shoe in either of the two locations. Looking only at shoe transactions (which is what we have direct access to), women remain in a location, 115 days on average. This likely underestimates how long they actually live in a location, because our data are both left and right truncated and we only examine shoe purchases, not all clothing purchases. This resulted in data from 2,007 individuals who placed 16,236 orders for shoes between the dates 6/14/2010 and 2/3/2015. Of those, 1,865 had data from areas where we were able to ascertain sufficient information (more on this below). Of those, 15 were outliers in terms of their number of moves (more than two standard deviations from the mean) and so were excluded, resulting in a final data set of 1,850 customers who placed 14,496 transactions (see Table 1 for summary statistics and S1 Fig for histogram of number of transactions per customer and S1 Table for correlation matrix of all relevant variables). Of note, the online retailer does not customize offerings by region within the United States, and therefore all shoe options were available to all individuals regardless of their geographic location.

**Table 1:** Select Descriptive Statistics.

General Information	Value			
Number of Unique Origin Locations	201			
Number of Unique Destination Locations	182			
Variable	Mean (StDev)	Min	Median	Max
Observations per Customer	1.52 (.95)	1	1	5
Observations per Origin Location	11.79 (40.04)	1	2	469
Observations per Destination Location	13.02 (37.22)	1	3	357
Origin				
Heel Size Purchased	1.64” (1.45”)	.13”	1.00”	5.75”
Norms (14 days)	1.63” (.61”)	.15”	1.61”	5.50”
MSRP	$220.62 ($144.79)	$1.00	$193.50	$1550.00
Population	18,886 (7,489)	238	20,283	66,830
Female %	51.3% (1.1%)	40.2%	51.4%	63.1%
Median Age	39.91 (3.98)	19.70	40.16	62.25
Median Household Income	$69,702 ($16,936)	$8,958	$72,118	$104,505
Education Years	13.27 (.86)	9.53	13.47	15.06
Destination				
Heel Size Purchased	1.90” (1.35”)	.13”	1.65”	6.00”
Norms (14 days)	1.96” (.45”)	.25”	1.95”	5.42”
MSRP	$251.67 ($166.61)	$1.00	$200.00	$1550.00
Population	19,102 (7,320)	157	20,283	66,830
Female %	51.2% (1.0%)	37.8%	51.3%	54.6%
Median Age	39.76 (3.99)	26.60	39.78	62.25
Median Household Income	$70,486 ($17,526)	$17,612	$72,118	$104,505
Education Years	13.29 (.90)	5.78	13.46	14.89

### Sales Transaction Variables

The dataset contains panel data at the transaction level. For each transaction, we observe unique user id, date of transaction, census region of transaction, MSRP (not the price paid) of shoe, number of shoes purchased in that location on that date, and heel size of shoe purchased. In addition to the transaction data, we constructed norm indices at the transaction level that allow us to identify the norms of the region that an individual purchased their shoes in. This was accomplished by averaging across all transactions of other customers in the region of interest for the 14 days prior to the transaction in question. That is, for each transaction, we constructed unique norm indices by averaging across all other transactions from other customers in the census region where the transaction took place for the previous 14 days. This allowed us to construct norm indices for heel size and MSRP. These norm indices are particularly useful as they naturally account for seasonal variations in norms (e.g. higher heels purchased in the summer time) because they only look at recent transactions.

In order to ascertain the impact of location on heel size preferences, we transform the data into individual level transaction data averaging across sales within a geographic region within an individual. We classify the data into two groups: data from the origin location and data from the destination location. Origin location data is the average of all transaction data from a given individual within the first observed location in the dataset. Destination location data is the average of all transaction data from a given individual within a subsequent location in the dataset. For each individual, there may be more than one such origin-destination pair (if someone moved multiple times) and so we include multiple rows of data as needed and control for both individual level variation and move number with random effects. See S2 Fig for a histogram of number of moves.

### Missing Data

Because some transactions were either 1) at the very beginning of our data collection window (left censored data) or 2) in geographic regions with no other transactions for the 14 days prior to the transaction in question, we were unable to construct norm data for them. There were 398 (2.7%) observations that had any such missing data. Because the type of data missing varied across customers, we do not exclude customers merely for having one missing data value. Rather, we exclude customers when the data missing applies to the model being tested (see bottom of Table 2 for Customer and Observation sample sizes in each model).

**Table 2:** Mixed Models Predicting Log(Avg Own Heel Size Purchased in New Location).

Fixed Effects	Model 1	Model 2	Model 3	Model 4	Model 5	Model 6^1^	Model 7^2^
Log (Avg Own Heel Size in Origin)	.202 (.018)***	.202 (.018)***	.202(.018)***		.202 (.018)***	.198 (.018)***	.203 (.020)***
Origin Norms	.049 (.031)	.047 (.032)	.045 (.032)		.080 (.034)*	-.022 (.082)	.087 (.036)*
Destination Norms	.300 (.042)***	.302 (.042) ***	.333 (.043) ***	.381 (.043)***	.280 (.045)***	.705 (.118)***	.265 (.054)***
Change in Median Income (1000s)		.001 (.001)	-.009 (.004)*	-.009 (.004)*	-.009 (.004)*	-.018 (.007)*	-.010 (.005)*
Destination Norms x Change in Median Income (1000s)			.005 (.002)*	.005 (.002)**	.005(.002)**	.011 (.004)**	.006 (.002)*
Change in Avg MSRP					.001 (.000)**	.001 (.001)	.001 (.000)**
Change in Population Size (1000s)					.001 (.002)	.000 (.002)	.003 (.002)
Change in % of Women					.511 (1.424)	.912 (1.420)	1.167 (1.550)
Change in Median Age					-.001 (.004)	-.000 (.004)	.001 (.004)
Change in # of Ed. Years					-.015 (.020)	-.009 (.020)	-.025 (.022)
**Random Effects**							
Customer	Yes	Yes	Yes	Yes	Yes	Yes	No
Move Number	Yes	Yes	Yes	Yes	Yes	Yes	No
Origin	Yes	Yes	Yes	Yes	Yes	Yes	Yes
Destination	Yes	Yes	Yes	Yes	Yes	Yes	Yes
Month	No	No	No	No	No	Yes	No
AIC	5726.2	5727.2	5722.8	5848.7	5704.9	5743.9	4715.7
Number of Customers^3^	1846	1846	1846	1851	1841	1849	1837
Number of Observations	2226	2226	2226	2235	2220	2233	1837

Note—Values in parentheses are standard errors.

* p < .05,

** p < 01,

*** p < .001

1—Model 6: Origin and Destination Norms and Change in Avg MSRP include all history of location, not just previous 14 days. 2—Model 7 uses only data from first move. 3—Number of Customers and Observations differ due to variations in missing data.

### Demographic Variables

The socioeconomic status of a geographic region was determined with 2010 ZIP code level US census data and create census region averages. Specifically, for each region, we observe the population size, % of population that is female, the median age of the individuals, average years of education of individuals, and, most critically, the median household income of individuals.

### Ethics Statement

The first author consulted the institutional review board of his university prior to the start of research. The review board deemed that this research does not require approval as the data being used are archival in nature and, critically, lack any identifiable information making them anonymous.

### Data Availability

The firm providing the data used in the subsequent analyses has requested that the data not be made publicly available. All authors signed non-disclosure agreements with the firm to such an effect. The firm has allowed aggregate data such as that reported in the Tables of this paper and any results from data analyses that do not inadvertently reveal individual level data to be made publicly available. For any additional aggregate analyses, the corresponding author can be contacted and aggregate results can be shared on an ad hoc basis.

## Results

### Main Effects of Relocation

Does purchase behavior after moving reflect conformity to the new location or consistency with their past behavior? To address this question, we ran a series of mixed model regressions with random intercepts for individual, move number, origin location, and destination location predicting own average heel size purchased in the destination location (Table 2). Of note, origin location and destination location random effects allow us to control for any variation in the general nature of the locations that we could not observe with region level data (e.g. New York City’s cutting edge fashion scene vs. Topeka, Kansas’ more conservative approach to fashion). Heel size was log transformed in order to normalize the data.

When predicting the average heel size purchased in the destination location as a function of the average heel size purchased in the origin location (a measure of personal preferences), origin norms, and destination norms (Model 1), own preferences from the origin location (*B* = .20, *SE* = .02, *t* = 10.97, *p* < .001) and destination norms (*B* = .30, *SE* = .04, *t* = 7.23, *p* < .001) significantly predict own preferences in the destination location, while origin norms have no predictive power (*B* = .05, *SE* = .03, *t* = 1.58, *p* = .12). In other words, behavior in a new location demonstrates both consistency and conformity, balancing past behavior with the influence of new norms. Interestingly, origin norms, though not significant direct predictors of destination behavior, do operate indirectly via established individual preferences (see S1 Appendix for an explanation).

### Moderation of Relocation Effects by Socioeconomic Status

**Fig 1:**
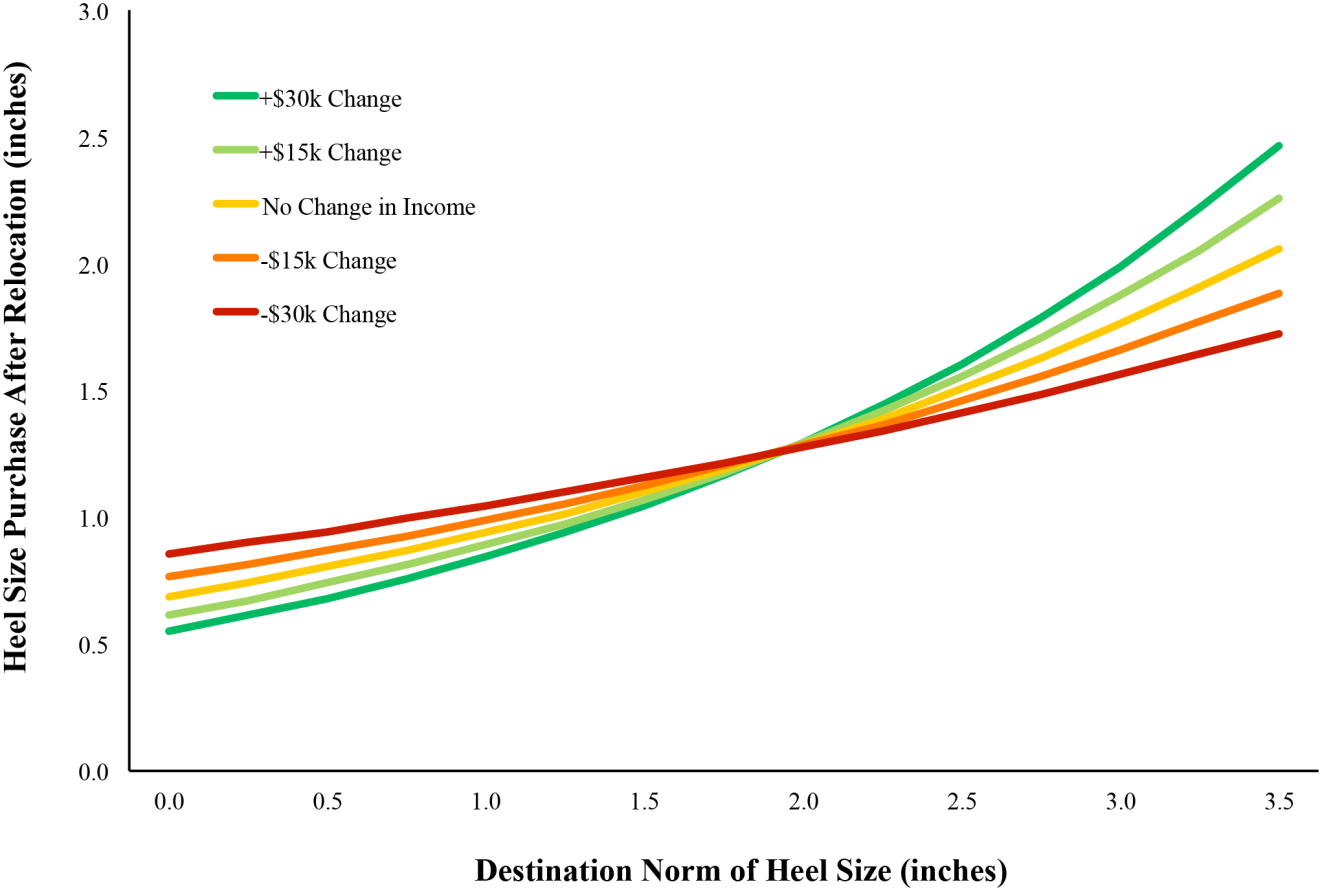
Heel-size purchase behavior across upward and downward relocations (each line represents a different change in median annual income). Estimates from full model (Model 4).

People who move may demonstrate some level of conformity, but it may differ between moves to higher SES locations (*i*.*e*., *upward relocations*) versus moves to lower SES locations (i.e., *downward relocations*). To test this, we computed a new variable—change in median income between origin and destination behavior—and tested whether this interacted with conformity level in the same mixed model (Model 3). As can be seen by the crossing lines in Fig 1, the analysis revealed a significant interaction (*B* = .005, *SE* = .002, t = 2.52, *p* = .01). Of note, to assure that the origin norms and origin behavior covariates do not bias our interaction estimate [20] we also test the interaction in Model 4 without these statistical controls. Doing so does not change our conclusion as the interaction term is of the same magnitude and still statistically significant (*B* = .005, *SE* = .002, t = 2.66, *p* = .007).

The influence of destination norms is largest when the change in median income between locations is large and positive, and smallest when the same change is large and negative. When an individual moves to a higher status location, their behavior strongly reflects the norms of that destination. However, when an individual moves to a lower (or similar) status location, they mostly do not assimilate to those new norms and instead remain relatively consistent in their preferences.

To give a sense of the size of this effect, we can consider an individual moving to a new location that has a median income level that is $22,723 *higher* (1 standard deviation, in terms of difference between destination and origin median income levels) than their origin location’s income. All else being equal, if the norms of that new destination are that shoes are purchased with an average heel height of 1”, then the behavior of this individual will be to purchase shoes with heel height of about .95”. Likewise, if the norms of that new destination are that shoes are purchased with an average heel height of 3”, then the behavior of this individual will be to purchase shoes with heels of about 2.11” in height. In other words, this person will largely assimilate to the preferences of their new location. In contrast, if the same person moved to a new location that has a median income level that is $22,723 *lower* than their origin location, all else being equal, their decision about heel height is far less influenced by the norms of that new location. For instance, if the average heel height preference of others in that new location is 1”, then the behavior of this individual will be to purchase shoes with heels that are about 1.14” in height. However, unlike when moving to a higher income level location, when the average heel height preference of others in this new location is 3”, then the behavior of this individual is largely unaffected by these norms and they purchase shoes that are about 1.56” in height. In other words, though there is some assimilation to this new higher heel norm, there is far less assimilation than when the new location is of a higher SES. Put another way, when moving to a new location with a median income that is $22,723 *higher* than the origin location, the unstandardized beta coefficient between destination norms and destination behavior is .54, whereas when moving to a new location with a median income that is $22,723 *lower* than the origin location, the same beta coefficient is only .21, a sign of far less conformity to new norms.

### Robustness checks

To test the robustness of the SES change by conformity interaction, we preform three checks. First (Model 5), we include a series of fixed effects controls: change in average MSRP of shoes across locations, change in population size across locations, change in % of women across locations, change in median age across locations, and change in number of years of education across locations. Even when including these controls, we observe a significant interaction between destination norms and change in median income between locations (*B* = .005, *SE* = .002, t = 2.61, *p* = .009).

Second, we change the nature of the norm variables to include averages across all previous transactions, not just those made in the previous 14 days (Model 6). Because this specification removes the benefit of seasonality being controlled for by the nature of how the norms variables were computed, we include a new random effect of Month. This ensures that any variations we observe cannot solely be attributed to seasonal differences. In Model 6, we still observe a significant interaction between destination norms and change in median income between locations (*B* = .011, *SE* = .004, t = 2.67, *p* = .007).

**Fig 2:**
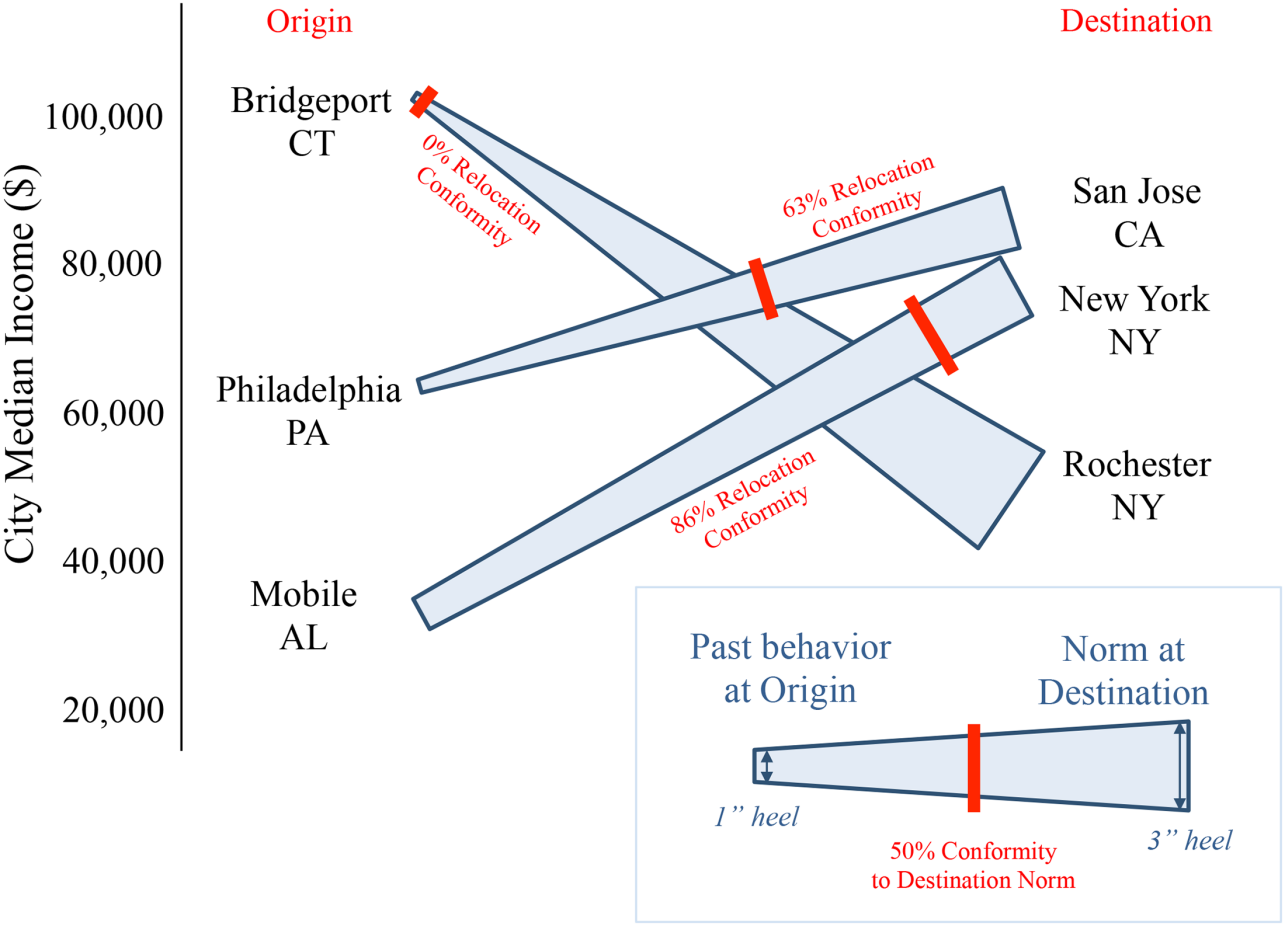
Three select behaviors for illustration purposes (each trapezium is an individual move).

Finally, it is possible that customers who move often exert excessive influence on our model and so even with the customer and move number random effects controls, our results are driven by these unusual individuals (Model 7). We reran our analysis with only the first observed move from each individual (removing the customer and move number random effects as they are no longer needed). Doing so once again yields a significant interaction between destination norms and change in median income between locations (*B* = .006, *SE* = .002, t = 2.45, *p* = .01). In sum, regardless of model specification, upward relocations involve greater conformity to destination norms than downward relocations, which involve greater consistency with origin behavior. This is true even when controlling for location factors including MSRP of shoes purchased, population size, average age, gender ratio, and education level. To make this complex relationship a bit clearer, we include three illustrative examples in Fig 2.

## General Discussion

These findings provide a large-scale demonstration of how people balance conformity and consistency in new environments based on upward versus downward socioeconomic transitions. People adopt the fashion trends of higher status locations to a far greater extant than they do lower status locations. These results represent an empirical demonstration of long held theories in sociology and pervasive lay beliefs.

Despite the robustness and geographic scope of this dataset, many questions remain. One question is whether this preferential conformity reflects self-distancing from low status groups [21], self-identification with high status groups [22], or both. Another outstanding question for future research is whether such conformity is conscious or non-conscious [23, 24]. Finally, recent work has demonstrated that conformity is more likely to occur when others explicitly express their preferences as compared to when they act on those preferences [25]. In the context of fashion, it seems more likely that the observed conformity occurred due to observation of norms, rather than conversations about preferences, suggesting that conformity is, indeed, influenced by observed preferences, at least in this one case. Future work is needed to better explain the inconsistency between our findings and those showing that conformity is less likely to occur when merely observing the preferences of others.

In addition to these questions, it is worth understanding whether status-dependent conformity effects emerge with other fashion choices, such as color, cut, or formality. Given the public nature of all such fashion choices, we suspect it would. In contrast, more private choices such as music or television selection may be less sensitive to conformity. Future research should examine the boundaries of trickle down preferences.

The non-experimental nature of the present work leaves open the possibility for alternative attributions for fashion choices. Although we included numerous control variables, it is possible that some unobserved third variable may help explain our results. Nevertheless, past experimental work provides converging evidence for our findings, as does recent network analyses. Our results are also sensitive to one other issue typically observed in these types of archival studies: reverse causation. However, it seems implausible that heel size is what drove relocation decisions rather than vice versa.

## Conclusion

Every introduction to psychology textbook includes a section on Asch’s famous conformity experiments and how such behavior is nearly universal [26]. Likewise, textbooks discuss the universality of consistency and cognitive dissonance [27]. However, there are times at which these theories make conflicting predictions, such as when people relocate. Our data suggest that people balance these psychological demands based on a powerful extrinsic variable—socio-economic status—causing people to conform upward more than downward. In this way, people may be able to ratchet themselves up the social ladder, one heel at a time.

## Supporting Information

S1 Appendix Supplementary Mediation Analysis. (DOCX)

S1 Fig Histogram of Number of Transactions per Customer. (DOCX)

S2 Fig Histogram of Number of Moves per Customer. (DOCX)

S1 Table Correlation Matrix. (DOCX)
