# Supplementary material for: Trickle-Down Preferences: Preferential Conformity to High Status Peers in Fashion Choices
Source: PLoS One. 2016 May 4;11(5):e0153448. doi: 10.1371/journal.pone.0153448 (PMC4856365; doi:10.1371/journal.pone.0153448)
Supplement: S1 Appendix — (DOCX) [file pone.0153448.s001.docx]

**S1 Appendix. Supplementary Mediation Analysis**

It is not the case that the origin norms have no predictive power. Indeed, as described in the main text, origin norms influence destination behavior through origin behavior. That is, though we don’t see an influence of origin norms on destination behavior in Model 1, a mediation analysis predicting destination behavior with origin behavior as the mediator, yields significant mediation. Specifically, following the recommendation of MacKinnon et al. (2002) we test mediation by taking the maximum of two specific p-values when comparing the following two models (with all random effect controls present):

1. Origin Behavior predicated by Origin Norms (p-value for the influence of Origin Norms)
2. Destination Behavior predicted by both Origin Norms and Origin Behavior (p-value for the influence of Origin Behavior on Destination Norms when controlling for Origin Norms).

Origin Norms significantly predict Destination Behavior (*B* = .13, *SE* = .03, *t* = 4.10, p < .001; Figure 1), and Origin Norms significantly predict Origin Behavior (*B* = .29, *SE* = .04, *t* = 8.14, *p* < .001). When Origin Norms and Origin Behavior are both used to predict Destination Behavior in the same model, Origin Norms predict Destination Behavior to a far smaller extent than in the previous model, (*B* = .07, *SE* = .03, *t* = 2.23, *p* = .03), and Origin Behavior strongly predicts Destination Behavior (*B* = .21, *SE* = .02, *t* = 11.48, *p* < .001). When taking the maximum of the two relevant p-values, we find that the mediation is significant (*p* < .001). In other words, origin norms influence origin behaviors, which, in turn, influence destination behavior.

Mediation of origin norms on destination preferences through origin behavior.

Origin Behavior

*B* = .29***

*B* = .21***

Origin Norms

Destination Behavior

*B* = .13*** , *B*’ = .07*

MacKinnon DP, Lockwood CM, Hoffman JM, West SG, Sheets V. A comparison of methods to test mediation and other intervening variable effects. Psychological methods. 2002 Mar;7(1):83.
