# Supplementary material for: Trickle-Down Preferences: Preferential Conformity to High Status Peers in Fashion Choices
Source: PLoS One. 2016 May 4;11(5):e0153448. doi: 10.1371/journal.pone.0153448 (PMC4856365; doi:10.1371/journal.pone.0153448)
Supplement: S1 Fig — (DOCX) [file pone.0153448.s002.docx]

**S1 Figure. Histogram of Number of Transactions per Custom**er
