# Supplementary material for: Trickle-Down Preferences: Preferential Conformity to High Status Peers in Fashion Choices
Source: PLoS One. 2016 May 4;11(5):e0153448. doi: 10.1371/journal.pone.0153448 (PMC4856365; doi:10.1371/journal.pone.0153448)
Supplement: S1 Table — (DOCX) [file pone.0153448.s004.docx]

**S1 Table. Correlation Matrix**

|  | 1 | 2 | 3 | 4 | 5 | 6 | 7 | 8 | 9 | 10 |
| --- | --- | --- | --- | --- | --- | --- | --- | --- | --- | --- |
| (1) Log(Avg Own Heel Size Purchased in New Location) | 1 |  |  |  |  |  |  |  |  |  |
| (2) Log(Avg Own Heel Size in Origin Location) | 0.25*** | 1 |  |  |  |  |  |  |  |  |
| (3) Origin Norms | 0.09*** | 0.18*** | 1 |  |  |  |  |  |  |  |
| (4) Destination Norms | 0.17*** | 0.10*** | 0.12*** | 1 |  |  |  |  |  |  |
| (5) Change in Median Household Income (1000s) | 0.01 | 0 | 0.06** | -0.07*** | 1 |  |  |  |  |  |
| (6) Change in MSRP | 0.10*** | -0.01 | -0.28*** | 0.34*** | 0.04 | 1 |  |  |  |  |
| (7) Change in Population Size (1000s) | 0.03 | 0.01 | -0.05* | 0.09*** | 0.11*** | 0.07** | 1 |  |  |  |
| (8) Change in Female % | 0.00 | -0.01 | 0.06* | -0.08*** | 0.29*** | 0.00 | 0.00 | 1 |  |  |
| (9) Change in Median Age | -0.01 | 0.02 | 0.04 | -0.05* | 0.27*** | 0.00 | -0.28*** | 0.38*** | 1 |  |
| (10) Change in Years of Education | -0.02 | -0.02 | 0.04* | -0.12*** | 0.55*** | -0.01 | -0.10*** | 0.30*** | 0.50*** | 1 |

* p < .05, ** p < 01, *** p <.001
